# Prevalence and psychological correlates of borderline personality traits among medical students in Egypt: a multicenter cross-sectional study

**DOI:** 10.1186/s12888-025-07514-8

**Published:** 2025-11-05

**Authors:** Yusof Mohamed Omar, Mazen Ghozzy, Youssef Eladawy, Farah Ahmed Elsayed, Maryam M. Abdelrady, Salma A. Wafa, Mohamad Kedan, Abdullah Elsaeid, Rahma M. Almetwaly, Mohamed Hossam Ghazy, Alaa Salah Abdelmawgoud

**Affiliations:** 1 Faculty of Medicine, Mansoura University, Mansoura, Egypt; 2 Mansoura Students’ Scientific Association, Faculty of Medicine, Mansoura University, Mansoura, Egypt; 3 Medical Program, Mansoura University Hospitals, Mansoura, Egypt; 4 Psychiatry Department, Faculty of Medicine, Mansoura University, Mansoura, Egypt

**Keywords:** Borderline personality disorder, Psychological distress, Screening, Medical students, Egypt

## Abstract

**Background:**

Borderline personality traits are prevalent among university students and can significantly impair their emotional and interpersonal functioning. This study aimed to assess the prevalence of borderline personality traits and their psychological correlates among medical students in Egypt.

**Methods:**

A multicenter cross-sectional study was conducted during the 2024/2025 academic year, involving 1,293 medical students from eight Egyptian universities. Borderline personality traits were screened using the McLean Screening Instrument for Borderline Personality Disorder (MSI-BPD), a self-report screening tool derived from the BPD module of the Diagnostic Interview for DSM-IV Personality Disorders. While anxiety symptoms were assessed with the Generalized Anxiety Disorder-2 (GAD-2), and depressive symptoms were assessed with the Patient Health Questionnaire-2 (PHQ-2), respectively. Pearson’s correlation and multivariable binary logistic regression analyses were performed to identify factors associated with borderline personality traits.

**Results:**

Participants reported an average of 3.85 traits on the MSI-BPD, with 18.5% screening positive for clinically significant borderline personality traits. The most prevalent symptoms were mood instability (53.2%) and chronic feelings of emptiness (47.0%), while 17.2% reported suicidal ideation. Correlation analyses showed significant positive relationships between borderline personality traits and both depression (*r* = 0.52, *p* < 0.001) and anxiety (*r* = 0.61, *p* < 0.001). Multivariable regression identified three significant predictors of positive borderline personality traits screening: family history of mental illness (Adjusted Odds Ratio (AOR) = 2.17, 95% CI: 1.57, 3.01), anxiety symptoms (AOR = 1.38, 95% CI: 1.25, 1.53), and depressive symptoms (AOR = 1.38, 95% CI: 1.24, 1.54).

**Conclusion:**

Borderline personality traits are prevalent among Egyptian medical students and are strongly associated with anxiety, depression, and familial mental health history. These findings highlight the need for early screening and targeted interventions to support students at risk.

**Clinical trial number:**

Not applicable.

## Introduction

Borderline Personality Disorder (BPD) is a complex and serious psychiatric condition characterized by a pervasive pattern of affective instability, distorted self-image, and tumultuous interpersonal relationships [1]. While BPD is formally diagnosed in clinical settings, many individuals, particularly young adults, may exhibit subthreshold features or traits associated with the disorder [2]. These traits, though less severe than full-syndrome BPD, are nonetheless clinically relevant and can significantly impair functioning [3]. Prevalence estimates of BP traits vary, but seem particularly pronounced in university populations [4], with some studies reporting rates as high as 41.2% [5], underscoring the importance of early recognition in non-clinical populations.

Etiological models of BPD highlight the dynamic interplay between genetic predisposition [6], early life adversity [7], and ongoing psychosocial stressors [8]. The transition to university life represents a critical developmental period marked by significant psychological demands, including separation from familiar support systems, heightened academic expectations, and the need for increased autonomy [9, 10]. These challenges are even more pronounced in medical education, where students are exposed to intense workloads, high-stakes assessments, and emotionally taxing clinical encounters [11].

The high-demand medical environment may contribute to factors associated with the development or exacerbation of borderline personality traits, such as maladaptive coping strategies, excessive striving for academic validation, and social isolation [12, 13]. These factors, in turn, contribute to emotional instability, impulsivity, and identity disturbance, which can compromise students’ ability to form stable relationships and maintain a coherent sense of self [14, 15]. In more severe cases, these manifestations may escalate to self-injury or suicidal ideation, with individuals exhibiting borderline personality traits facing a risk of suicide attempts many times that of the general population [16].

Given that medical students consistently report elevated levels of psychological distress, including anxiety, depression, and suicidality, which may co-occur with or be exacerbated by underlying BPD symptomatology [17–19], there is a pressing need for early screening of borderline personality traits in this population. Early identification of borderline personality traits is critical, as timely intervention can reduce symptom progression, mitigate comorbid mental health conditions, and improve long-term psychosocial outcomes [20]. However, despite the clear clinical significance of borderline personality traits and their consequences, research on their prevalence and correlates remains limited among medical students, particularly in low- and middle-income countries such as Egypt.

Accordingly, this study was designed with two primary objectives. First, we aimed to establish the point prevalence of clinically significant borderline personality traits among medical students in Egypt, addressing a key gap in the regional literature. Second, we sought to explore the psychological correlates of these traits. Specifically, we aimed to examine the association between borderline personality trait scores and symptom severity of both anxiety and depression. We further aimed to identify potential predictors for screening positive, investigating factors such as family history of mental illness, demographic characteristics, and psychological distress markers. By fulfilling these objectives, our findings can better inform early screening strategies and contribute to the development of tailored mental health interventions.

## Methods

### Study design and period

A descriptive multi-centric cross-sectional study with an analytical component to explore associations between borderline personality traits and other psychological distress markers was conducted during the academic year 2024/2025.

### Study sample and settings

The study sample consisted of first- to fifth-year medical students from eight universities across three of Egypt’s official administrative and geographic regions. This national classification system includes the Urban Governorates (major metropolitan areas), Lower Egypt (the Nile Delta region north of Cairo), and Upper Egypt (the Nile Valley region south of Cairo). The included institutions were Cairo and Alexandria Universities (representing the Urban Governorates region); Mansoura, Tanta, and Zagazig Universities (representing the Lower Egypt Governorates region); and Souhag, Qena, and Al-Fayoum Universities (representing the Upper Egypt Governorates region). No medical schools from the Frontier Governorates region were included, as they were recently founded and did not offer a full five-year academic program at the time of data collection.

### Sample size

The sample size was calculated using the OpenEpi calculator. Based on an expected prevalence of 41.2% for clinically significant borderline personality traits among university students reported in a study from Saudi Arabia [5], a 95% confidence interval, and a 5% margin of error, the required sample size was estimated at 372 medical students. To account for the convenience sampling technique and regional variability, a design effect of 3 was applied, resulting in a final target sample size of 1,116 medical students. Ultimately, 1,293 participants completed the questionnaire.

### Sampling method and data collection approach

A convenience sampling approach was utilized after determining the required sample size. Using Google Forms, respondents completed the questionnaire. Data was collected from first-year to fifth-year students across universities. The questionnaire was delivered to all students through official groups via the Telegram app and other social media platforms. They could answer anonymously, on their own time, and were not compensated. At each university, a team of collaborators was assembled and instructed by the authors on how to approach students online. Students who didn’t consent to participate, students with incomplete responses, and interns were excluded from the study.

### Study tool

The questionnaire consisted of four sections, each measuring a specific variable of interest.

The first section collected sociodemographic data, including sex, age, academic year, self-reported residential environment (urban vs. rural), and family history of mental disorders.

Borderline personality traits were assessed using the McLean Screening Instrument for Borderline Personality Disorder (MSI-BPD) [21], a self-report screening tool derived from the BPD module of the Diagnostic Interview for DSM-IV Personality Disorders [22]. The MSI-BPD consists of 10 yes/no items, covering nine DSM-IV-TR BPD criteria (with two items addressing the paranoia/dissociation criterion). A score of 7 or more positive responses serves as the screening threshold, indicating likely BPD symptoms warranting further clinical evaluation. The MSI-BPD demonstrates strong psychometric properties, with a sensitivity of 81% and specificity of 85% in the general population. Diagnostic efficiency improves further in younger individuals, with sensitivity reaching 90% and specificity 93% for those under 25 [21].

Anxiety symptoms were assessed using the Generalized Anxiety Disorder 2-item scale (GAD-2), a brief self-report screening tool derived from the GAD-7, designed to assess core anxiety symptoms [23]. It consists of two questions: “Over the last 2 weeks, how often have you been bothered by the following problems? 1) Feeling nervous, anxious, or on edge; 2) Not being able to stop or control worrying.” Responses are rated on a 4-point Likert scale from 0 (“not at all”) to 3 (“nearly every day”), yielding a total score between 0 and 6. A score of 3 or higher is commonly used as a cut-off to indicate clinically significant anxiety symptoms. The GAD-2 demonstrates strong psychometric properties, with a sensitivity of approximately 86% and specificity of 83% for detecting generalized anxiety disorder [24].

Depressive symptoms were assessed using the Patient Health Questionnaire 2-item scale (PHQ-2), a brief self-report screening tool derived from the PHQ-9, designed to assess core depressive symptoms [25]. It consists of two questions: “Over the last 2 weeks, how often have you been bothered by the following problems? 1) Little interest or pleasure in doing things; 2) Feeling down, depressed, or hopeless.” Responses are rated on a 4-point Likert scale from 0 (“not at all”) to 3 (“nearly every day”), yielding a total score between 0 and 6. A score of 2 or higher is commonly used as a cut-off to indicate clinically significant depressive symptoms. The PHQ-2 demonstrates strong psychometric properties, with a sensitivity of approximately 89.3% and specificity of 75.9% for detecting major depressive disorder [26].

### Statistical analysis

Data were analyzed using Jamovi software version 2.3.28.Categorical data were presented as frequencies and percentages, whereas continuous data were presented as means and standard deviations (SD). The normality of continuous variables was assessed visually using Quantile-Quantile (Q-Q) plots. Pearson correlation coefficients were used to examine relationships between continuous variables, including age, PHQ-2 scores, GAD-2 scores, and MSI-BPD scores. Univariable and multivariable binary logistic regression analysis was conducted to identify predictors of dichotomized clinically significant borderline personality traits screening status. A p-value less than 0.05 was deemed statistically significant.

## Results

### Sociodemographic and mental health characteristics of participants

A total of 1,293 medical students participated in the survey, with a nearly equal distribution by gender (52.0% female). The largest proportion of participants were in their 4th academic year (35.5%), while the smallest group was in the 1st year (13.8%). Most participants resided in urban areas (74.2%), with the largest regional representation from urban governorates (42.3%). Approximately 29.1% of students reported a family history of mental illness (Table 1).

**Table 1:** Sociodemographic and mental health characteristics of the study participants (*N* = 1293)

Characteristics		Total *n* (%)
Age	Less than 21	422 (32.7%)
	21 and older	870 (67.3%)
Sex	Female	672 (52.0%)
	Male	620 (48.0%)
Academic year	1st year	178 (13.8%)
	2nd year	192 (14.9%)
	3rd year	220 (17.0%)
	4th year	459 (35.5%)
	5th year	243 (18.8%)
Region	Urban Governorates	547 (42.3%)
	Lower Egypt Governorates	464 (35.9%)
	Upper Egypt Governorates	281 (21.7%)
Residence	Urban	959 (74.2%)
	Rural	333 (25.8%)
Family history of mental illness	No	916 (70.9%)
	Yes	376 (29.1%)
Positive screening for BPD ^1^	No	1053 (81.5%)
	Yes	239 (18.5%)
Mental health screening results^2^	Neither condition	634 (49.1%)
	Depression only	133 (10.3%)
	Anxiety only	189 (14.6%)
	Both depression and anxiety	336 (26.0%)

^1^Screening via MSI-BPD (≥ 7); ^2^Screening via PHQ-2 (depression) and GAD-2 (anxiety) (≥ 3)

18.5% of students met the screening threshold. Additionally, 10.3% screened positive for depression only, 14.6% for anxiety only, and 26.0% for both anxiety and depression.

### Frequencies of borderline personality traits

Table 2 displays the frequency of endorsement for each item of the MSI-BPD. The most commonly reported traits included “being extremely moody” (53.2%), “feeling chronically empty” (47.0%), and “feeling angry or acting in an angry manner” (49.5%). Items related to self-harm (17.2%) and impulsivity (24.8%) were among the least frequently endorsed. The two items corresponding to DSM-IV Criterion 9 (paranoid ideation or dissociation) were endorsed by 48.1% and 34.1% of participants, respectively. The overall mean MSI-BPD score was 3.85 (SD = 2.7) (Table 3).

**Table 2:** Frequency of medical students in Egypt responses to MSI-BPD items (*N* = 1293)

MSI-BPD item	DSM-IV criteria	Total Yes *n* (%)
Have you made desperate efforts to avoid feeling abandoned or being abandoned?	Criterion 1: Frantic efforts to avoid real or imagined abandonment	384 (29.7%)
Have any of your closest relationships been troubled by a lot of arguments or repeated breakups?	Criterion 2: A pattern of unstable and intense interpersonal relationships	577 (44.7%)
Have you often felt that you had no idea of who you are or that you have no identity?	Criterion 3: Identity disturbance	470 (36.4%)
Have you had at least two other problems with impulsivity?	Criterion 4: Impulsivity in at least two areas that are potentially self-damaging	321 (24.8%)
Have you deliberately hurt yourself physically? How about made a suicide attempt?	Criterion 5: Recurrent suicidal behavior, gestures, or self-mutilating behavior	222 (17.2%)
Have you been extremely moody?	Criterion 6: Affective instability due to a marked reactivity of mood	687 (53.2%)
Have you chronically felt empty?	Criterion 7: Chronic feelings of emptiness	607 (47.0%)
Have you felt very angry a lot of the time? How about often acted in an angry or sarcastic manner?	Criterion 8: Inappropriate, intense anger or difficulty controlling anger	640 (49.5%)
Have you often been distrustful of other people?	Criterion 9: Transient, stress-related paranoid ideation or severe dissociative symptoms	622 (48.1%)
Have you frequently felt unreal or as if things around you were unreal?		441 (34.1%)

Abbreviations: DSM IV = Diagnostic and Statistical Manual of Mental Disorders IV, MSI-BPD = McLean Screening Instrument for BPD

**Table 3:** Pearson correlation coefficients between MSI-BPD scores, PHQ-2 scores, GAD-2 scores and participant age

	MSI-BPD	GAD-2	PHQ-2
Age	-0.060*	-0.011	-0.044*
PHQ-2	0.517**	0.608**	—
GAD-2	0.606**	—	—
Mean (SD)	3.85 (2.7)	2.25 (1.79)	2.42 (1.73)

Abbreviations: GAD-2 = Generalized Anxiety Disorder-2; MSI-BPD = McLean Screening Instrument for BPD, PHQ-2 = Patient Health Questionnaire-2; SD = Standard Deviation; *p-value = < 0.05, **p-value = < 0.001

### Correlations between mental health measures

Pearson correlation analysis (Table 3) revealed moderate positive correlations between MSI-BPD scores and both PHQ-2 depression scores (*r* = 0.517, *p* < 0.001) and GAD-2 anxiety scores (*r* = 0.606, *p* < 0.001). A weak but statistically significant negative correlation was observed between age and MSI-BPD scores (*r* = − 0.060, *p* = 0.045).

### Factors associated with screening positive for clinically significant borderline personality traits

Univariable binary logistic regression analysis (Table 4) showed that students in the 2nd academic year had higher odds of screening positive for clinically significant borderline personality traits compared to 1st-year students (Crude Odds Ratio (COR) = 1.70, 95% CI: 1.04, 2.77, *p* = 0.033). Students residing in rural areas had lower odds of screening positive (COR = 0.70, 95% CI: 0.50, 0.98, *p* = 0.040). A family history of mental illness was strongly associated with increased odds of screening positive (COR = 3.01, 95% CI: 2.25, 4.02, *p* < 0.001). Higher GAD-2 anxiety scores (COR = 1.68, 95% CI: 1.54, 1.83, *p* < 0.001) and PHQ-2 depression scores (COR = 1.72, 95% CI: 1.57, 1.87, *p* < 0.001) were also significantly associated with greater odds of screening positive.

**Table 4:** Univariate and multivariate linear regression of MSI-BPD scores among medical students in Egypt

Variable		Univariate regression		Multivariate regression	
		COR (95% CI)	*p*-value	AOR (95% CI)	*p*-value
Age (continuous)		0.96 (0.88–1.05)	0.356	1.11 (0.94–1.32)	0.217
Sex	Female	1.00	—	1.00	—
	Male	0.80 (0.60–1.06)	0.126	1.14 (0.82–1.59)	0.442
Academic year	1st year	1.00	—	1.00	—
	2nd year	1.70 (1.04–2.77)	0.033	1.68 (0.94–3.01)	0.079
	3rd year	0.91 (0.55–1.52)	0.725	0.81 (0.41–1.59)	0.533
	4th year	0.69 (0.43–1.08)	0.107	0.55 (0.27–1.14)	0.107
	5th year	1.02 (0.62–1.66)	0.951	0.69 (0.29–1.67)	0.415
Region	Urban Governorates	1.00	—	1.00	—
	Lower Egypt Governorates	0.83 (0.60–1.14)	0.238	0.96 (0.65–1.42)	0.844
	Upper Egypt Governorates	0.89 (0.62–1.29)	0.540	0.74 (0.48–1.13)	0.162
Residence	Urban	1.00	—	1.00	—
	Rural	0.70 (0.50–0.98)	0.040	0.88 (0.59–1.32)	0.537
Family history of mental illness	Not present	1.00	—	1.00	—
	Present	3.01 (2.25–4.02)	< 0.001	2.17 (1.57–3.01)	< 0.001
Anxiety score (GAD-2)		1.68 (1.54–1.83)	< 0.001	1.38 (1.25–1.53)	< 0.001
Depression score (PHQ-2)		1.72 (1.57–1.87)	< 0.001	1.38 (1.24–1.54)	< 0.001

Abbreviations: AOR = Adjusted Odds Ratio; COR = Crude Odds Ratio; CI = Confidence Interval; GAD-2 = Generalized Anxiety Disorder-2; MSI-BPD = McLean Screening Instrument for Borderline Personality Disorder; PHQ-2 = Patient Health Questionnaire-2

In the multivariable binary logistic regression model, after adjusting for other variables, family history of mental illness (Adjusted Odds Ratio (AOR) = 2.17, 95% CI: 1.57, 3.01, *p* < 0.001), GAD-2 score (AOR = 1.38, 95% CI: 1.25, 1.53, *p* < 0.001), and PHQ-2 score (AOR = 1.38, 95% CI: 1.24, 1.54, *p* < 0.001) remained significant independent predictors of screening positive for clinically significant borderline personality traits.

## Discussion

Despite increasing awareness of mental health challenges faced by medical students worldwide, there remains limited research focusing specifically on borderline personality traits and their correlates within this vulnerable population in Egypt. Early identification and screening for such symptoms are crucial for timely intervention, which can improve students’ wellbeing and academic performance. To address this need, the present study aimed to estimate the prevalence of borderline personality traits and to identify key associated factors among medical students in Egypt.

In our study, 18.5% of medical students screened positive for clinically significant borderline personality traits, a figure that falls within the wide global range reported in the meta-analysis by Meaney et al. [4], which found prevalence estimates ranging from 0.5% to 32.1%, depending on country and methodology. In contrast, substantially higher rates have been reported in some countries, such as Pakistan [27], where Naeem et al. found a prevalence of 62% using the Borderline Personality Inventory (BPI), and Saudi Arabia [5], where Alfakeh et al. reported a prevalence of 41.2% using the MSI-BPD.

These differences in prevalence may be attributed to methodological, cultural, and contextual variations. For instance, the BPI used in the Pakistani study was developed based on Kernberg’s psychodynamic theory of BPD [28, 29], whereas our study employed the MSI-BPD, a screening tool derived from the BPD module of the Diagnostic Interview for DSM-IV Personality Disorders [21, 22]. Such differences in conceptualization and operationalization of BPD have been highlighted by Meaney et al. [4], who emphasized the need for greater consistency in measurement tools across studies. In their review, they noted the use of diverse constructs such as traits versus symptoms across different instruments, which complicates comparisons and hinders accurate identification. These inconsistencies present a significant barrier to early detection and the effective implementation of support strategies for individuals exhibiting borderline personality traits.

Meanwhile, the significantly higher prevalence of borderline personality traits reported in the Saudi Arabian study may be attributed to contextual and cultural factors. In that study, 43.6% of university students reported experiencing adverse childhood events, and 61.4% of those who screened positive for borderline personality traits had a history of childhood trauma [5]. Additionally, over 90% of participants were still living with their families at the time of the study, which may have intensified the impact of highly controlling, unstable, or emotionally volatile family environments, which are known to contribute to the development and exacerbation of borderline personality traits [30]. These observations resonate with our own findings, where students with a family history of mental illness had significantly higher odds of screening positive for clinically significant borderline personality traits. Beyond the well-established hereditary component [6], this may also reflect the influence of shared family environments where emotional invalidation, inconsistency, or mental health stigma are present. Such dynamics may help explain the high endorsement of symptoms like chronic feelings of emptiness and identity disturbance among our participants, as these traits are often shaped by inconsistent emotional support, invalidation, or unclear role modeling within the family context [31].

In our study, students with higher levels of anxiety and depressive symptoms were significantly more likely to screen positive for clinically significant borderline personality traits, reinforcing the well-documented emotional dysregulation that characterizes the disorder [32, 33]. These overlapping symptoms can obscure the diagnosis, as emotional distress may be misattributed solely to mood or anxiety disorders rather than recognized as part of a broader personality pathology. This has particular relevance in the Egyptian context, where psychological distress among university students, particularly medical students, has reached concerning levels. At Ain Shams University, 64.2% of students screened positive for depression and 77.1% for anxiety [34], while a similar study in Upper Egypt reported prevalence rates of 65% and 73%, respectively [35]. Moreover, although our reported prevalence of suicidal ideation was 17.2%, previous research has found that 35.3% of Egyptian undergraduates have experienced such thoughts [36], underscoring a pervasive mental health crisis in this population.

Furthermore, these symptoms rarely occur in isolation. A previous study among Egyptian medical students reported elevated maladaptive personality domains, such as negative affect, detachment, and psychoticism, which negatively correlated with depression, suggesting a complex interplay between affective symptoms and underlying personality disturbances [37]. Interestingly, suicidal behavior (17.2%) and impulsivity (24.8%) were less frequently reported, indicating that psychological distress in this population may often be internalized. This aligns with previous findings showing that many Egyptian undergraduates are reluctant to seek mental health support due to discomfort discussing emotions [38], and express disappointment with the current mental health care system in Egypt [39], highlighting significant barriers to effective support.

Such silent suffering, combined with high emotional reactivity, places students at increased risk for persistent psychopathology and underscores the critical need for early identification and support. Altogether, these findings make a compelling case for integrating routine psychological screening into university health systems, particularly in medical schools where students may be at heightened risk. Proactive screening efforts that consider familial mental health history, trauma exposure, and co-occurring anxiety and depression could enable earlier recognition of borderline personality traits and timely intervention, potentially preventing the progression of distress into more entrenched psychopathology. Given the stigma and systemic barriers to care in Egyptian settings [36, 38, 40], such initiatives are not only recommended but urgently needed.

### Limitations

This study, while offering valuable insights into the prevalence and correlates of borderline personality traits among medical students, is subject to several limitations.

Firstly, its cross-sectional design prevents the establishment of causal relationships. Future longitudinal studies are therefore essential to track the trajectory of these traits over time and to better understand the temporal relationship between BPD traits, academic stress, and the onset of mood and anxiety disorders. Secondly, the reliance on a convenience sampling technique limits the generalizability of our findings. Subsequent research should employ probability-based sampling methods, such as stratified random sampling across different universities, to obtain a more representative national estimate. Furthermore, data collection through self-report questionnaires introduces potential social desirability and selection biases. Future work could mitigate these by using mixed-methods designs that combine anonymous surveys with qualitative interviews to explore lived experiences in greater depth. The use of screening instruments also means our findings represent likely traits rather than definitive clinical diagnoses. A crucial next step is to conduct two-stage studies that use these screening tools for initial case-finding, followed by structured clinical interviews to confirm diagnoses and establish a more accurate clinical prevalence. Finally, our exclusive focus on medical students means findings may not be transferable. Comparative studies are warranted to investigate whether this high prevalence is unique to the medical school environment or is similarly present among students in other demanding disciplines.

## Conclusion

This study highlights a notable prevalence of clinically significant borderline personality traits among medical students in Egypt, often co-occurring with heightened anxiety and depressive symptoms. Our findings underscore the significant burden of psychological distress within this demanding academic population and reinforce the complex interplay between emotional dysregulation and other common mental health concerns. These results call for increased awareness and proactive screening for borderline personality traits in medical education settings, particularly given the already concerning levels of mental health challenges observed in the region. Early identification and targeted interventions are crucial to improving the well-being and academic functioning of future healthcare professionals. While this study provides a critical foundation, the path forward requires more robust longitudinal and comparative research, using definitive diagnostic methods, to fully map the trajectory of these traits and develop effective, targeted interventions for this high-risk population.

## Data Availability

The data of this study is available from the corresponding author upon reasonable request.

## Declarations

### BPD Consortium

Mohamed Ashraf Elsaadany^1^, Mey E Bastawi^1^, Merna Ebrahim Abdelwahab^1^, Mayar Ahmed Zaki^1^, Nada Abbas^1^, Mohamed Khairy Ayoub^1^, Maram Mohamed Hassan^1^, Alaa Mahmoud Khedr^1^, Mahmoud Hekal^1^, Gannah Sherif^1^, Judy Abo El Seoud^1^, Yusuf Aboelazm Elba^1^, Reem Ashraf Mohamed^1^, Yousef Madkour Abo Al Ezz^1^, Youssef Mostafa Ashosh^1^, Amr Ahmed Abdo^1^, Tasbeeh Samy^1^, Leena Mohammed Saeed^1^, Thomas Emad Gergis^1^, Abdelrahman Seddik^1^, Kareem Monsef^1^, Mouhamed Elfagal^1^, Mohamed Zakaria Elnagdy^1^, Nourhan Gaber^1^, Moaz Osama^1^, Raneem Shoaib^1^, Sohila ElShabrawy^1^, Omar Elsayed Abdelkader Mohammed^1^, Amr Taher Massoud^1^

^1^Faculty of Medicine, Mansoura University, Mansoura, Egypt

## Abbreviations

AOR: Adjusted Odds Ratio
BPD: Borderline Personality Disorder
BPI: Borderline Personality Inventory
CI: Confidence Interval
COR: Crude Odds Ratio
DSM–IV: Diagnostic and Statistical Manual of Mental Disorders, Fourth Edition
GAD–2: Generalized Anxiety Disorder–2
MSI–BPD: McLean Screening Instrument for Borderline Personality Disorder
PHQ–2: Patient Health Questionnaire–2
Q–Q: Quantile–Quantile
SD: Standard Deviation
